# *Helicobacter pylori* Immune Response in Children Versus Adults

**DOI:** 10.18103/mra.v10i12.3370

**Published:** 2022-11-30

**Authors:** Victor E. Reyes

**Affiliations:** 1 Department of Pediatrics, Department of Microbiology and Immunology, University of Texas Medical Branch, 301 University Blvd. Galveston, TX 77555-0372 USA

## Abstract

*H. pylori* is perhaps the most prevalent human pathogen worldwide and infects almost half of the world’s population. Despite the decreasing prevalence of infection overall, it is significant in developing countries. Most infections are acquired in childhood and persist for a lifetime unless treated. Children are often asymptomatic and often develop a tolerogenic immune response that includes T regulatory cells and their products, immunosuppressive cytokines, such as interleukin (IL)-10, and transforming growth factor-β (TGF-β). This contrasts to the gastric immune response seen in *H. pylori*-infected adults, where the response is mainly inflammatory, with predominant Th1 and Th17 cells, as well as, inflammatory cytokines, such as TNF-α, IFN-γ, IL-1, IL-6, IL-8, and IL-17. Therefore, compared to adults, infected children generally have limited gastric inflammation and peptic ulcer disease. *H. pylori* surreptitiously subverts immune defenses to persist in the human gastric mucosa for decades. The chronic infection might result in clinically significant diseases in adults, such as peptic ulcer disease, gastric adenocarcinoma, and mucosa-associated lymphoid tissue lymphoma. This review compares the infection in children and adults and highlights the *H. pylori* virulence mechanisms responsible for the pathogenesis and immune evasion.

## I. INTRODUCTION

*Helicobacter pylori (H. pylori)* infection remains a considerable healthcare burden and is a prevailing member of the human gastric microbiome in ~50% of the world’s population. *H. pylori* is prolific in the USA among underrepresented minorities, the poor, and the elderly^1^, but is more prevalent in developing countries. Infection with *H. pylori* is most often seen during childhood^2^. The clinical presentation and gastric immune response it elicits in pediatric patients differs from that in adults. Although the frequency of pediatric infections is high, studies on *H. pylori* and host interactions in children are limited. The infection rates are higher in some children, primarily those from low socioeconomic backgrounds^3^. Since the initial observations by Robin Warren and Barry Marshall, who isolated the bacteria from gastric biopsies^4^, *H. pylori* infection is now linked to clinically significant outcomes, such as chronic gastritis, peptic ulcer or gastric malignancy [mucosa-associated lymphoid tissue (MALT) lymphoma and gastric adenocarcinoma]. Gastric cancer is a leading cause of cancer-related deaths claiming nearly one million lives annually (http://www.iarc.fr). Because of its association with gastric cancer, *H. pylori* is now classified as a class I carcinogen. Thus, *H. pylori* has important public health implications, and despite its high prevalence and clinical importance, there is no preventive or therapeutic vaccine against *H. pylori*. Multiple independent studies have reported on *H. pylori’*s ability to siege the host immune response; however, our understanding of how the mechanisms utilized by *H. pylori* to co-opt host immune defenses remains an important gap in the field, which has prevented the development of an effective vaccine. The mechanisms used by *H. pylori* to induce mostly T_reg_ cells in the gastric mucosa of children, and therefore, create a tolerogenic niche necessary for the bacteria to establish a lifelong infection remain unclear.

## II. EPIDEMIOLOGY

*H. pylori* bacteria persistently infects 4.4 billion individuals worldwide^1^, with a prevalence that varies widely between regions and countries. The highest rates occur in Africa, Latin America, the Caribbean, and Asia^5^. Although *H. pylori* infection is decreasing in the United States, gastric cancer is still an important cause of morbidity and mortality among underrepresented minorities^6^, disproportionally affected by *H. pylori*^7^. Hispanics have a higher prevalence of *H. pylori* infection than non-Hispanic whites (NHW; 30.3% vs. 9.2%).^8^ *H. pylori* infections predominantly occur during early childhood^9^ and persist for years or even decades, often without clinical signs. The infection is transmitted from person to person via the fecal-oral, gastro-oral, and oral-oral routes^10,11^. Intrafamilial transmission is an important aspect that has been examined by genotyping fecal samples^12^. Interestingly, the transmission of *H. pylori* from mother to child appears to be the most probable route of intrafamilial transmission. Waterborne transmission of *H. pylori* has also been documented^13^. A study in Japan showed that the prevalence of *H. pylori* was associated with water contamination in wells with *H. pylori*^14^. However, a study in Peru that sought to detect the presence of *H. pylori* in the tap water in homes of gastric cancer patients found no correlation between gastric infection and water contamination^15^. The investigators in that study found *H. pylori* in tap-water samples, but the rates of *H. pylori* detection were lower than in gastric cancer samples.

## III. *H. pylori* INFECTION IN CHILDREN

*H. pylori* infections are primarily acquired in childhood and persist for a lifetime unless treated. In most cases the infection is asymptomatic, and complications are less common in pediatric patients. A recent study estimated that about one-third of children worldwide are infected with *H. pylori*^16^, confirmed by a meta-analysis examining the prevalence of *H. pylori* infection in children younger than 18 years of age^17^. That study concluded that the incidence of *H. pylori* infection was significantly higher in low-income to middle-income countries than in higher-income countries, reflecting similar observations made in adults. Low socioeconomic status is the main predisposing factor for pediatric *H. pylori* infection^18^ and more common among those living in crowded dwellings^19^. Thus, despite global trends toward lower infection rates, *H. pylori* is still very common in children.

A study in Japan that included 332 patients aged 2–18 years showed that the most common clinical manifestations associated with *H. pylori* infection were gastritis, iron deficiency anemia, and duodenal ulcers, which represented 75% of those studied. Gastric ulcers were observed in <10% of all cases^20^. A link between gastric cancer and *H. pylori* infection in pediatric populations is unclear. Case reports of gastric malignancy in children infected with *H. pylori* are scarce. The few reported cases include a family history of gastric cancer, which may be related to a genetic predisposition. An intriguing observation is that allergic disorders and *H. pylori* seem to have an inverse association. Chen and Blaser initially reported an inverse association between *H. pylori* seropositivity and asthma in children^21^. Those observations were recently repeated by Fouda, *et al*. 2018, who also noted that *H. pylori* seropositivity safeguards against childhood asthma and is inversely correlated to its clinical and functional severity^22^.

Several studies have noted that *H pylori-*infected children have reduced gastric inflammation compared to the infected adults, despite comparable *H. pylori* colonization levels. Sequencing of the bacteria isolated from the infected children and adults showed similarity in *cagA* and *vacA* gene profiles, suggesting that variations in bacterial strains and key virulence factors could not account for the lower levels of inflammation in infected children^23^. A study by Harris *et al*. 2007, conducted with 36 children and 79 adults with abdominal symptoms in Chile showed that the level of gastritis in children was substantially lower than in adults^24^. In that study, the investigators observed that gastric levels of TGF-β1 and IL-10 and the frequency of T_reg_ cells in *H. pylori-*infected children are higher than in adults. The same investigators also reported that *H. pylori*-infected children have lower gastric IL-17-specific mRNA and protein levels and fewer gastric Th17 cells^23^. Together, these observations suggested a skewed CD4^+^ response toward a more tolerogenic gastric environment with increased T_reg_ cells and reduced mucosal Th17 response in infected children. Additionally, the gastric mucosa of the infected children has lower levels of IFN-γ mRNA, suggesting a diminished Th1 response in children with *H. pylori* infection. Interestingly, similar observations were made in mice^25^. Adult mice infected with CagA^+^ *H. pylori* promptly develop gastritis, gastric atrophy, epithelial hyperplasia, and metaplasia. In contrast, neonatal mice infected with the same *H. pylori* strain produced a tolerogenic response and were protected from preneoplastic lesions. These differences in the responses elicited by *H. pylori* in the mucosa of young subjects could explain why children are considerably less prone to develop significant clinical gastric diseases than *H. pylori-*infected adults.

## IV. *H. pylori* CHARACTERISTICS AND VIRULENCE MECHANISMS

*H. pylori* is a human-specific, spiral-shaped, flagellated, Gram-negative bacterium that selectively colonizes the gastric mucosa. *H. pylori* has an assortment of virulence factors that have been identified to play a role in the development of adverse outcomes associated with *H. pylori* infection. Some virulence factors aid *H. pylori* in adapting to the harsh environment within the gastric niche and facilitate chronic colonization by evading immune-mediated clearance, as discussed below.

### A. CagA

Among the virulence factors expressed by *H. pylori,* there are two toxins, the cancer-associated gene toxin (CagA) and the vacuolating cytotoxin (VacA), which are both critical in the pathology associated with infection and immune evasion. CagA is a cytotoxin encoded within an island of genes in a 40 kbp DNA segment and referred to as the *H. pylori cag* pathogenicity island, *cag-*PAI, possibly the most studied *H. pylori* virulence factor. The *cag*-PAI comprises a cluster of 31 genes, most of which code for a type 4 secretion system, T4SS. The T4SS is a syringe-like structure used by *H. pylori* to penetrate the gastric epithelial cell (GEC) membrane and translocate *H. pylori* products into the cytosol of GECs. CagA is one of the products injected and is the effector protein encoded at one end of *cag-*PAI and does not appear to have homologs in other bacterial species. However, type 4 secretion systems are expressed by several species of bacteria but are less complex.^26^ CagA is possibly the most virulent factor connected with *H. pylori* and is a risk factor for peptic ulcer disease and gastric cancer. In fact, CagA is considered an oncoprotein because it affects tumor suppressor signaling pathways via various molecular mechanisms and promotes neoplasia.

After CagA is translocated into GECs, it localizes to the inner leaflet of their plasma membrane, where it is phosphorylated at tyrosine residues within Glu-Pro-Ile-Tyr-Ala (EPIYA) motifs in the C-terminus by Src and Abl kinases^27^. Phosphorylated CagA initiates a series of signaling events. Four major EPIYA motifs (A, B, C, and D) are based on the amino acid sequence bordering the EPIYA motif on both sides. EPIYA-A, EPIYA-B, and EPIYA-C motifs in tandem are expressed by “western strains.” In contrast, “East-Asian strains” have CagA with EPIYA-A, EPIYA-B, and EPIYA-D motifs^28^. These motifs contribute to the polymorphism in the C-terminus of the protein and occur as tandem repeats varying in number from one to seven. The number of EPIYA motifs is relative to the extent of phosphorylation and the effects in epithelial cells. Src family kinases and c-Abl kinase phosphorylate the EPIYA motifs^29^. The phosphorylation of these EPIYA motifs plays a key role in neoplasia as demonstrated by studies in transgenic mice expressing phosphorylation resistant CagA^30^. In those studies, transgenic mice expressing wild-type *cagA* spontaneously developed gastrointestinal carcinomas and hematopoietic malignancies^30^. *H. pylori* CagA also promotes PD-L1 (B7-H1) expression by GECs, which could allow for immune avoidance by developing cancer cells^31^.

In addition to the CagA protein, the T4SS also translocates bacterial cell wall components such as peptidoglycan or muropeptides into GECs. *H. pylori* peptidoglycan is recognized by NOD1, an intracellular pathogen-associated molecular pattern (PAMP) recognition receptor that senses peptidoglycan. NOD1 activation leads to NF-κB activation and upregulation of proinflammatory immune responses. Peptidoglycan and *H. pylori* CagA injected into GECs lead to a reduced expression of B7-H2, a positive costimulatory of effector T lymphocytes, by activating the p70 S6 kinase pathway^32^.

CagL is also a *cag-*PAI-encoded protein. CagL is a pilus structure component that develops at the interface between *H. pylori* and GECs. Significantly, CagL enhances the binding of the T4SS to α5β1 integrin receptor on GECs. CagL possesses an arginine-glycine-aspartate (RGD) motif that is a recognition site for integrins^33^. Deletion of CagL abolishes *H. pylori*’s ability to stimulate IL-8 secretion by GECs, suggesting that CagL is essential for the translocation of CagA by the T4SS. Other Cag proteins, such as CagY, CagI, and CagA, may also bind to integrin. This binding results in cellular alterations, such as cell spreading, focal adhesion formation, and tyrosine kinases’ activation.

### B. VacA

Another major *H. pylori* virulence factor is the vacuole-inducing cytotoxin (VacA), a key secreted protein without a known homolog in other bacterial species. It contributes to gastric colonization and the pathogenesis of gastric neoplasia. VacA is initially synthesized as a 140 kDa pro-toxin, including an N-terminal signal peptide, a central region representing the toxin, and a C-terminal domain participating in transport function. Following processing, the central region (~88 kDa) representing the mature virulent form of the toxin is secreted and processed further into two subunits of 33 kDa (A subunit) and 55 kDa (B subunit)^34^, or remains on the bacterial surface^35^. The p33 form was initially regarded as a pore-forming subunit, while the p55 form was initially viewed as the cell-binding component. However, both subunits are known to contribute to binding and vacuole formation. The exact entry mechanism is still in question as various receptors have been proposed but binding to sphingomyelin appears to be important in the process.

Although all strains of *H. pylori* have the *vacA* gene, there is a great deal of diversity in the gene, which includes three regions: signal- (s), mid-(m), and intermediate (i)-regions^36^. There are two allelic types for each region. Most virulent strains have the s1, i1, and m1 alleles associated with the highest risk of gastric adenocarcinoma.^37^ While s1 forms of VacA induce vacuoles, type s2 forms of VacA do not have that property. This is because different signal sequence cleavage sites in s1 and s2 VacA proteins affect the vacuolating ability of the toxin.^36^ The increased risk of disease for *H. pylori* strains containing s1, i1, or m1 forms of *vacA* is likely due to the coexpression of additional virulence factors. For instance, type s1 *vacA* allele-containing strains usually include *cag*-PAI^38^.

VacA has pleiotropic effects on host cells^39^. The most studied is its ability to induce vacuole formation that results in the disruption of endosomal trafficking^40,41^. This effect on endosomes, in turn, impairs the processing and presentation of foreign antigens^42^. A closely related property of VacA is its ability to induce autophagy, a process that depends on VacA binding to low-density lipoprotein receptor-related protein 1 (LRP1)^43^. *H. pylori* VacA has also been reported to alter host cell mitochondria and cell signaling^44,45^, disrupt epithelial barriers^46^, and cause cell death via apoptosis and necrosis^47,48^. Among the effects of VacA on other cells is the ability to impair T cell responses^49^, which is likely a mechanism that aids *H. pylori* in immune evasion properties, as discussed below.

### C. Urease

The urease enzyme is produced in large amounts by *H. pylori* and is directly linked with virulence. *H. pylori* urease is probably the most abundant protein produced by *H. pylori,* representing ten percent of the total protein^50^. Urease stimulates the rise of gastric pH through urea hydrolysis leading to the production of CO_2_ and ammonia, which help neutralize gastric acidity. This effect on gastric pH critically contributes to the colonization of *H. pylori* and its pathogenesis. The importance of urease in successful colonization was demonstrated when mutant strains lacking urease could not establish persistent infection^51–53^. The urease expression by *H. pylori* has been used to aid clinical diagnosis by developing various rapid urease tests to detect *H. pylori* in gastric specimens. Since CO_2_ is a product of urease hydrolysis of urea, a breath test was developed that employs either ^13^C or ^14^C-labeled urea. The subject ingests the labeled urea, and the tagged CO_2_ in the breath is measured using a detector.

*H. pylori* urease is comprised of two proteins, α and β subunits^54^. The α subunit (UreA) is approximately 30 kDa and the β subunit (UreB) is 60 kDa. Six of each subunit contribute to forming a dodecamer of about 600 kDa. Urease is found both inside and outside *H. pylori*. Approximately 30% of *H. pylori* urease is localized on the surface of intact cells after lysis of neighboring bacteria^55,56^. The urease expression outside the bacteria allows it to display biological activity independent of its enzymatic action. *H. pylori* urease has been shown to bind to class II MHC molecules and CD74 on GECs^57–60^. The interaction of urease with these cell surface proteins results in the induction of proinflammatory cytokines and apoptosis of host cells^60,61^. *H. pylori* urease was also reported to activate neutrophils and prevents their apoptosis^62^. Recently, *H. pylori* urease was shown to have proangiogenic activity both *in vitro* and *in vivo*^63^. This biological activity could be significant in gastric neoplasia associated with the infection. Another recent study showed that *H. pylori* urease might also bind to toll-like receptor (TLR)-2 on GECs, stimulating their expression of the human transcription factor hypoxia-induced factor-1α (HIF-1α). Notably, enzymatic activity was not required for this response^64^. In a previous study, these investigators showed that HIF-1α activation occurs via a PI3K-dependent pathway to create a G0/G1 cell cycle arrest in GECs^65^. The investigators in that study highlighted that the urease/TLR2/HIF-1 axis in immune cells was related to the generation of tolerance and posited whether activation of this axis in GECs might also play a role in the development of pre-neoplastic lesions^64^.

*H. pylori* expresses other virulence factors that play an essential role in shaping the immune response and modulating the immune microenvironment to favor persistent infection. For clarity, those bacterial components will be discussed below in the context of immune evasion mechanisms.

## V. IMMUNE EVASION PROPERTIES

Multiple studies have documented the remarkable ability of *H. pylori* to siege the host immune defenses surreptitiously. *H. pylori* has an array of evasion pathways to successfully escape innate and adaptive immunity to persistently infect the gastric mucosa and cause diverse gastrointestinal conditions.

### A. Innate Immunity

#### 1. Pattern recognition receptors

Pattern recognition receptors (PRRs) play a crucial role in innate immunity. PRRs recognize pathogen-associated molecular patterns (PAMPs) or molecules secreted by damaged cells (damage-associated molecular patterns (DAMPs)). PRRs induce various downstream signaling pathways essential for pathogen clearance upon their activation. Four families of PRRs have been described that include Toll-like receptors (TLRs), NOD-like receptors (NLRs), RIG-like receptors (RLRs), and C-type lectin receptors (CLRs)^66^. These receptors are strategically localized in the cell to allow recognition of conserved molecular structures of pathogens with diverse life cycles. GECs and immune cells in the lamina propria express TLRs that recognize various *H. pylori* PAMPs. TLR2 and TLR4 recognize lipopolysaccharide (LPS), TLR5 flagellin, and TLR9 CpG motifs in bacterial DNA. Yet, *H. pylori* effectively evade recognition by these TLRs through structural modification of the corresponding PAMPs^67–69^.

#### 2. Phagocytes

Macrophages and neutrophils are essential elements of innate immunity as they are responsible for the phagocytosis of bacteria. However, *H. pylori* possess antiphagocytic activity^70^. In a report where *H. pylori* phagocytosis by human and murine macrophages was monitored with immune fluorescence and electron microscopy, *H. pylori* strains that were *cag*-PAI^+^ and VacA^+^ avoided intracellular killing by hindering actin polymerization and phagosome formation^71^. The phagosomes that contained *H. pylori* formed clusters and fused, forming “megasomes” containing numerous bacteria, which allowed resistance to intracellular killing. However, a subsequent study reported that the fusion of those phagosomes was independent of VacA and CagA^72^. *H. pylori* bacteria have yet another mechanism to escape intracellular killing. *H. pylori* expresses catalase and superoxide dismutase that detoxify reactive oxygen species (ROS) and protect *H. pylori* from ROS^73,74^. *H. pylori* also downregulates CXCR1 and CXCR2 expression in human neutrophils, which are receptors to IL-8, which is the neutrophil recruiting chemokine^75^. This effect of *H. pylori* on CXCR1 and CXCR2 limits neutrophil migration and reduces bacterial killing. Studies by Gobert *et al*., 2001 have shown that *H. pylori* arginase competes with iNOS in macrophages for the substrate, L-arginine, and induces the expression of arginase II (Arg2)^76,77^. These two mechanisms protect *H. pylori* from NO-mediated killing.

#### 3. Dendritic Cells

Dendritic cells (DCs) represent critical mediators of innate and adaptive immunity since they capture antigens and present them to T cells. *H. pylori* promotes a “tolerogenic” phenotype in DCs. *H. pylori* inhibited DC maturation in response to LPS and a panel of other inducers of DC maturation when naïve DCs were co-cultured with *H. pylori*^78^. Ann Müller, *et al*. 2013, demonstrated that *H. pylori* reprograms DCs toward a tolerogenic phenotype since they did not elicit T effector cell responses^78^. Instead, DCs that were exposed to *H. pylori* induced in naïve T cells the expression of the transcription factor forkhead box protein 3 (FOXP3), which is the master regulator of T regulatory (T_regs_) cells. The induction of T_regs_ by *H. pylori*-exposed DCs depended on IL-18 signaling since T_regs_ did not develop if IL-18^−/−^ BM-DCs or T cells lacking the IL-18 receptor were used.

### B. Adaptive Immunity

#### 1. Humoral Immunity

Although *H. pylori* elicit a robust adaptive immune response, studies that examined humoral immunity in *H. pylori*-infected persons showed that although they produce *H. pylori-*specific IgA and IgG, the antibodies do not control *H. pylori*. However, differences in the antibody response were noted between subjects who developed gastritis or duodenal ulcers compared to subjects who developed gastric cancer^79^. The serum antibody titers in infected individuals who developed gastritis or duodenal ulcers demonstrated a higher IgG response than in those who developed gastric cancer. In contrast, gastric cancer patients displayed a more robust IgA titer than subjects with gastritis and duodenal ulcers. A separate study showed that a weak antibody response was associated with a high risk of developing gastric cancer in infected individuals^80^.

#### 2. Cell-Mediated Immunity

##### a. Th1 Cells.

The T cell response to *H. pylori* includes activation of both CD4^+^ and CD8^+^ T cells since both infiltrate the *H. pylori-*infected gastric mucosa. We and others reported that the response is polarized to Th1 cells^81,82^, which are ineffective in protecting against extracellular pathogens, such as *H. pylori*. This was an early clue that *H. pylori* surreptitiously maneuvers the host response to establish persistent infection. The *H. pylori* neutrophil-activating protein (*HP*-NAP) is an essential mediator in this response. *HP*-NAP has been shown to act on neutrophils and monocytes, causing them to secrete IL-12 and IL-23, and these cytokines foster Th1 responses^83^. Adding *HP*-NAP to T cell lines promoted a shift from a predominant Th2 to a Th1 phenotype of the T cell lines. The polarizing property of *HP*-NAP in these studies was shown when *HP*-NAP redirected allergen-induced T cell lines to express a Th1 cytokine profile instead of the characteristic Th2 cytokine profile induced in allergen responses^83^. Although Th1 cells may afford some protection from *H. pylori* by limiting its growth, they also seem to aid in pathogenesis, as supported by studies in human carriers suggesting Th1 participation in *H. pylori*-associated lesions. The Th1 cytokine IFNγ is linked to damaging effects associated with *H. pylori* infection by aiding inflammatory processes that result in gastritis and gastric neoplasia^84^. IFNγ directly induces inflammatory mediators and apoptosis of GECs^85,86^. Recent studies in a mouse model of autoimmune gastritis showed that IFN-γ^−/−^ mice had almost complete abrogation of precancerous histopathological atrophy and metaplasia compared to IFN-γ-sufficient controls^85^.

##### b. T regulatory Cells.

In addition to Th1 cells, other CD4^+^ T cell subsets infiltrate the lamina propria of the gastric mucosa in *H. pylori-*infected patients. Those CD4^+^ T cells include T_reg_ and Th17 cells. We and others noted the presence of Th17 and T_reg_ cells in the infected gastric mucosa^87–93^. T_reg_ cells play a role in the maintenance of peripheral self-tolerance^91^. T_reg_ cells inhibit T effector cells using various mechanisms, including cell-cell contact and immunosuppressive cytokines: TGF-β, IL-10, and IL-35.^91^ There are two subclasses of T_reg_ cells: natural T_reg_ (nT_reg_) and inducible T_reg_ (iT_reg_) cells, and both express the master transcription factor FOXP3, encoded by the *foxp3* gene on the X chromosome^94–96^. The nT_reg_ cells develop in the thymus, while antigenic stimulation of naive CD4^+^T cells in the presence of TGF-β and IL-2 triggers T_reg_ cell differentiation in peripheral lymphoid organs^91^. The presence of T_reg_ cells in gastric tissue biopsies from infected persons is well documented^88–90^. The stimulation of T_reg_ cells in the gastric mucosa of *H. pylori*-infected persons may explain early reports of hyporesponsiveness by T cells from *H. pylori*-infected subjects when restimulated with *H. pylori* antigens compared to T cells from uninfected persons^97^. An interesting observation pertinent to the Th1 cell response elicited by *H. pylori* is that those Th1 cells can be reprogrammed into T_reg_ cells^98^. Despite the robust inflammation induced by *H. pylori*, bacterial clearance is often inadequate due to activated T_reg_ cells. It is important to note that the gastric epithelium is instrumental in expanding T_reg_ cells. GECs respond to *H. pylori* infection with the expression of B7-H1^99,100^, discussed in detail below, and TGFβ^92^ and it is important to note that both of these proteins promote the development of T_reg_ cells^99,100^. The expansion of T_reg_ cells in the *H. pylori-*infected gastric mucosa could promote persistent infection and limit tissue damage associated with an excessive inflammatory response. This is supported by observations in *H. pylori*-infected mice deficient in T_reg_ cells, which developed increased pathology^101^.

##### c. Th17 Cells.

Th17 cells represent a CD4^+^ T cell subset important in immune protection against extracellular bacteria. Th17 cells are a proinflammatory CD4^+^ T cells subset that arises from antigen stimulation of naïve CD4^+^ T cells in the presence of TGF-β and IL-6, but IL-1β and IL-23 are also crucial in driving Th17 maturation. These cytokines are produced during *H. pylori* infection^102,103^. IL-23 promotes the maintenance and pathogenicity of Th17 cells by inhibiting Tbet and FOXP3, master regulators for Th1 and T_reg_ cells, respectively^104,105^. Interestingly, when CD4^+^ T cells from *H. pylori*-infected mice were co-cultured with macrophages in the presence of the *H. pylori* UreB subunit, that led to the induction of Th17 cells and production of IL-17A. Immunization with recombinant UreB induced UreB-specific Th17 cells and reduced *H. pylori* numbers.^106^ The differentiation of Th17 cells is regulated by the transcription factors retinoic acid receptor-related orphan receptors (ROR)γt and RORα^107,108^. Deficiency of RORγt or RORα impairs the differentiation of Th17 cells^107^. Pinchuk *et al.,* 2013, showed that gastric stroma, specifically myofibroblasts, induced Th17 cells during *H. pylori* chronic infection and gastric cancer^109^. Gastric myofibroblasts isolated from *H. pylori*-infected biopsies or resected gastric tissue when co-cultured with naïve CD4^+^ T cells led to the expression of RORγt and IL-17 by CD4^+^ T cells. As might have been predicted, the process was IL-6, TGF-β, and IL-21 dependent^109^. Th17 cells are known to produce a battery of proinflammatory cytokines that include IL-17A, IL-17F, IL-21, IL-22, and IL-26^110^. The cytokines produced by Th17 cells promote neutrophil recruitment and secretion of antimicrobial peptides. IL-17 has pleiotropic effects as it may mediate antibacterial action and may also have a pathogenic outcome. Mouse immunization studies showed that Th17 cells protect against *H. pylori* by limiting bacterial growth^111,112^, but in infected mice, *H. pylori* impair Th17 cells by inhibiting their costimulation and tips the balance to T_reg_ cells^32^. The T_reg_/Th17 balance is essential to immune homeostasis. The role that IL-17A and IL-17F play in *H. pylori* infection in humans is unclear, and studies suggest that these cytokines may contribute to pathology. Various studies with Asian patients have identified that genetic variants of IL-17 are associated with a risk of gastric cancer^113^. Still, several studies comparing circulating levels of IL-17 in gastric cancer patients versus healthy subjects have had conflicting results, possibly due to the inclusion of different populations in the studies.

##### d. Th2 and Th22 Cells.

Other CD4^+^ T cell subsets have been examined in the context of *H. pylori* infection, but their role is unclear. Th2 cells are not highly activated within the *H. pylori*-infected mucosa but have been considered protective due to observations in mice orally immunized with recombinant *H. pylori* urease B subunit plus cholera toxin^114^. The immunization led to the development of a Th2 cell response that caused a progressive reduction in IFN-γ and the clearance of *H. pylori*. However, a subsequent study by Garhart, *et al*., 2003, found that immunized double-knockout in IL-4 and IL-5 mice were protected from the *H. pylori* challenge^115^, which suggested that Th2 cells were not necessary for protection. Th22 cells represent a newer CD4^+^ T cell subset that is understudied in the context of *H. pylori* infection. Perhaps due to their similarities with Th17 cells concerning IL-22 production, expression IL-22 was previously ascribed to Th17 cells. But it is now accepted that both Th17 and Th22 subsets produce IL-22, and the latter produces the highest levels of IL-22. Th22 cells are now recognized as a critical source of IL-22 and are found at the infection site and in various inflammatory conditions. A recent study that included 47 patients with peptic ulcer disease and 48 uninfected subjects found that Th22 cell numbers and IL-22 expression in the infected subjects were significantly more than in uninfected subjects^116^. Also, both parameters in infected subjects with PUD were significantly greater than in the infected subjects with gastritis. In addition, the investigators noted an inverse correlation with the T_reg_ counts in the infected subjects with PUD and gastritis.

##### e. CD8 T Cells.

CD8^+^ T cells have been detected in the *H. pylori*-infected gastric mucosa^117^. Studies on human CD8 gastric T cells are limited, perhaps because *H. pylori* is primarily an extracellular bacterium. In one study, investigators examined proliferation and IFN-γ production by circulating CD4^+^ and CD8^+^ T cells from infected and uninfected subjects in response to *in vitro* stimulation with *Helicobacter* antigens^118^. They observed modest proliferation by both T cell subsets from both groups. However, *H. pylori* antigens induced IFN-γ production, preferentially by CD8^+^ cells. Since recent studies have detected *H. pylori* inside epithelial cells, there may be more interest in follow-up studies on the role of gastric CD8^+^ T cells in *H. pylori* immunopathogenesis^119^.

#### 3. Inhibition of T cells.

The activation of T cells requires recognition by their antigen receptor (TcR) of peptides/MHC complexes expressed on antigen-presenting cells (APCs) and a second signal delivered by costimulatory molecules on the APCs. *H. pylori* uses multiple mechanisms to interfere with multiple steps leading to T-cell activation or viability. Some mechanisms involve the disruption of APC functions. *H. pylori* may prevent or delay phagocytosis by macrophages^120,121^. *H. pylori* bacteria that are internalized induce phagosome fusion into compartments that are referred to as megasomes^120^. As stated earlier, the VacA toxin disrupts endosomal traffic. It thus prevents antigen processing.^122^ Gebert *et al*., 2003, showed that VacA also acts directly on T cells by inhibiting signaling and proliferation through the induction of a G1/S cell cycle arrest^123^. This effect resulted from the interference with T cell receptor/IL-2 signaling at the level of the Ca^2+^-calmodulin-dependent phosphatase calcineurin. The translocation of the nuclear factor of activated T cells (NFAT), a transcription factor that regulates immune response genes, into the nucleus was abolished, causing downregulation of IL-2 transcription^123^. However, a subsequent independent study showed that human T cell proliferation inhibition was not attributable to VacA effects on NFAT activation or IL-2 secretion. Instead, the inhibition of T cell proliferation by VacA entails an intact N-terminal hydrophobic region required for forming anion-selective membrane channels that prevent the clonal expansion of T cells already activated by *H. pylori* antigens^49^. The *cag-*PAI, described above, induced apoptosis of T cells via a process highly dependent on the induction of Fas ligand (FasL) by *cag* PAI-bearing strains^124^. However, a separate report showed that the mitochondrial pathway mediates T cell apoptosis^125^. In that study, *H. pylori*-induced apoptosis of T cell lines was not blocked by inhibition of the death ligands TRAIL (TNF-related apoptosis-inducing ligand), FasL, and TNF-α.

Another mechanism that *H. pylori* use to inhibit T cells directly is gamma-glutamyl transpeptidase (GGT). GGT is a 60 kDa proenzyme that, after catalysis, yields a heterodimer of 40 kDa and 20 kDa subunits^126^. It is a threonine N-terminal nucleophile (Ntn) hydrolase that catalyzes the transpeptidation and hydrolysis of the gamma-glutamyl group of glutathione and also has an affinity for glutamine. GGT converts glutathione into glutamate and cysteinyl glycine, while its action on glutamine produces glutamate and ammonia. It is secreted by *H. pylori* and induces cycle arrest in lymphocytes in the G1 phase^127^. Schmees, *et al*.,2007, showed that the G1 arrest was due to disruption of Ras- and not PI3K-dependent signaling by *H. pylori* GGT and inhibited T cell proliferation. *H. pylori* arginase is another *H. pylori* enzyme that prevents T cell proliferation by depleting L-arginine availability^128^. The role of *H. pylori* arginase is to hydrolyze L-arginine to urea and ornithine. Urea is converted by urease to ammonia, which neutralizes gastric pH. *H. pylori* arginase lowers the expression of the CD3ζ-chain of the T cell receptor (TCR), which is critical for T cell activation. Arginase inhibitors reverted the effect, and an isogenic arginase mutant strain of *H. pylori* did not alter T cell function^128^.

#### 4. Immune Checkpoints.

**Figure 1.**
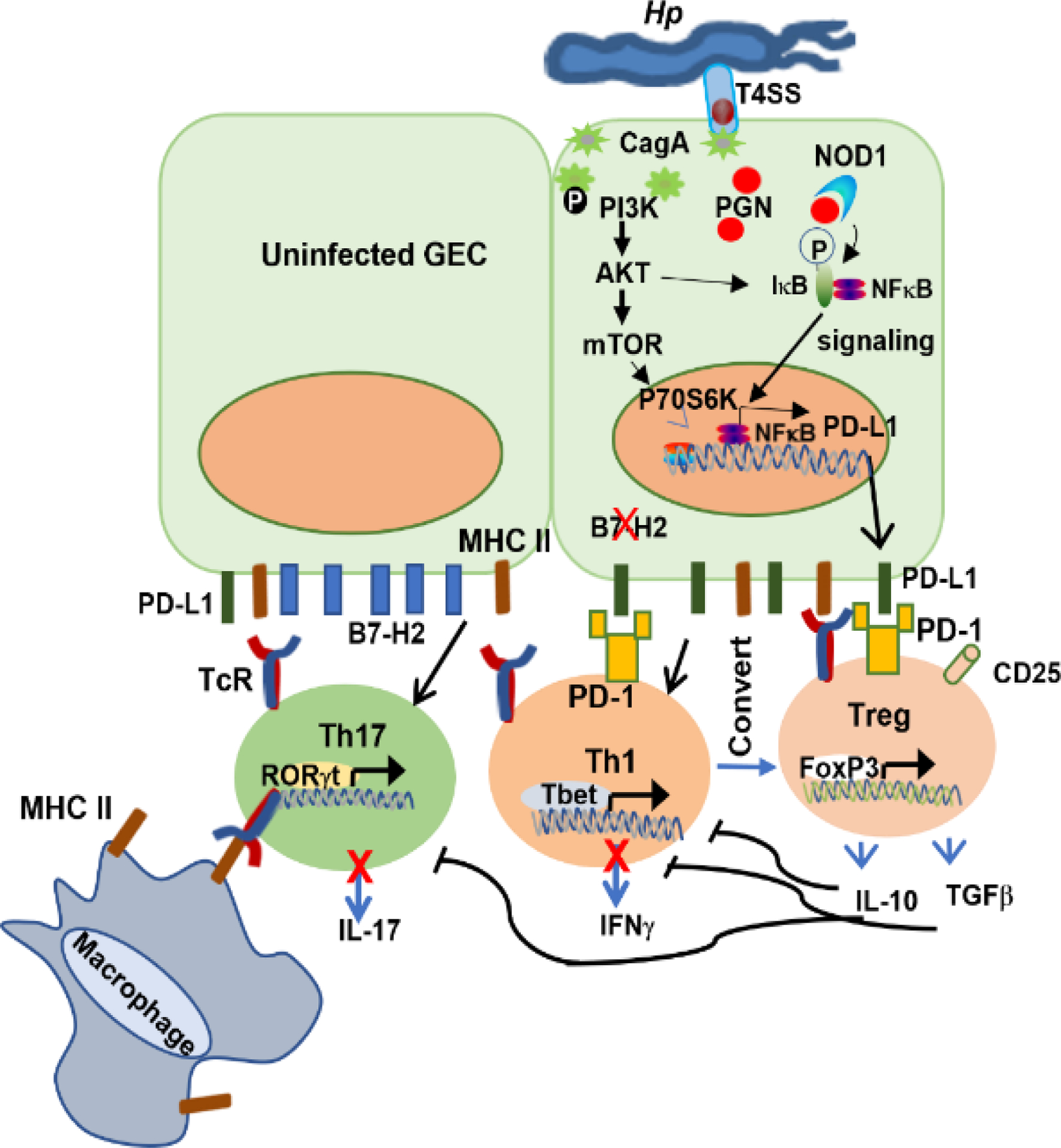
Model of the cross-talk between *H. pylori*, GECs and immune cells in the LP. *H. pylori* activates mechanisms that alter expression of immune checkpoints PD-L1 (increase) and B7-H2 (decrease). Altered expression of these receptors have consequences on T cells leading to suppressed or skewed T cell responses that allow *H. pylori* immune escape and chronicity. CagA activates multiple signaling pathways, including the PI3K/AKT/mTOR pathway that affects immune checkpoint expression.

The absence of the second signal during recognition by T cells of antigen-MHC complexes renders T cells anergic. The costimulatory signals delivered by members of the B7 family of receptors are balanced by coinhibitory signals that limit the extent of T cell activity and whose absence may lead to uncontrolled T cell proliferation^129^. B7 family ligands are a group of proteins initially represented by B7–1 (CD80) and B7–2 (CD86), whose interactions with CD28 and CTLA4 begin and end T cell activation, respectively^130–132^. This family of regulatory receptors has grown to include PD-L1 (B7-H1), PD-L2 (B7-DC), ICOS-L (B7-H2), B7-H3, and B7-H4 (B7-S1). More recently, B7-H5 (Vista)^133^, B7-H6^134^, and B7–H7 (originally called HHLA2)^135,136^ emerged; however, their expression patterns, ligands, and functions are still being defined. PD-L1, PD-L2, and ICOS-L were initially defined for T cell activation or tolerance through binding their coreceptors PD-1 (for PD-L1 and PDL2) and ICOS (for ICOS-L) on activated and memory T cells^137–139^. The B7 family of costimulatory/coinhibitory receptors has emerged as pivotal in immune regulation, maintaining a delicate balance between immune potency and suppression of autoimmunity (reviewed in^140–142^). Therefore, this family of receptors is now collectively known as immune checkpoints, and the pioneers who described them were the recipients of the 2018 Nobel prize in Physiology and Medicine. As reviewed previously^143^, these proteins act as rheostats for T cell activity. Studies suggest their role in influencing T cell differentiation or phenotype. Interestingly, *H. pylori* have mechanisms that allow the bacteria to control the expression of these immune checkpoints^143,144^. For instance, we demonstrated that *H. pylori* elicit heightened expression of PD-L1 (aka CD274, B7-H1) by GECs *in vitro* and *in vivo*^31,99^. PD-L1 expression by GECs from biopsies of *H. pylori*-infected subjects is significantly greater than on GECs from uninfected subjects, and infection of GECs induced the expression of PD-L1^99^. This feature of *H. pylori* is important because PD-L1 stifles the proliferation of effector CD4^+^ T cells^99^ and promotes the development of T_reg_ from naïve CD4^+^ T cells^100^(Figure 1). One study showed that PD-L1 converted Tbet^+^ Th1 cells into FOXP3^+^ T_reg_ cells *in vivo*^145^, which results in impaired cell-mediated immunity and shed some light on the previously unrecognized plasticity of T cells. Some recent studies redefined the functions of immune checkpoints as being able to fine-tune T cell responses, shape T cell phenotypes, or reprogram “terminally differentiated” T cell subsets^100,145–148^.

The mechanisms regulating this family of receptors’ expression are under active study due to their potential in immunotherapeutics. In fact, immune checkpoint inhibitors have become an important pillar in cancer immunotherapy, allowing long-term survival in patients with metastatic disease. However, *H. pylori* exploits those regulatory mechanisms to promote its persistence in the host. In addition to inducing PD-L1 expression by GECs^99,100^, *H. pylori* also induce the expression of B7-H3^149^, a receptor with dual function. While causing increased expression of co-inhibitors, *H. pylori* simultaneously prevent the expression of B7-H2^32^ (aka CD275, ICOS-L, B7RP-1), which is the only positive costimulator known to act on activated or memory T cells. Thus, *H. pylori* sets a “perfect storm” that prevents host effector T cells from clearing the infection. These responses partially depend on *H. pylori* CagA and peptidoglycan translocated by the type 4 secretion system (T4SS).

A recent study by Amieva’s group used high-resolution mapping that led to development of a model to explain how *H. pylori* establish persistent infection in the stomach by colonizing microniches deep in the gastric glands^150^. They found that a low number of bacterial founders at first establish colonies deep in the gastric glands and later grow and colonize adjacent glands, producing clonal population islands that persist. Interestingly, their work tied together previous observations regarding the age of the infected subject and T cell immunity on how they influence the outcome of the host-bacterial interactions since they noted that both factors regulate bacterial density within the glands.

## VI. CONCLUSIONS

Globally, infection with *H. pylori* remains a challenge in the pediatric population as it is in this age group when most cases initially become established via the oral route and persist for a lifetime. Through thousands of years of coexistence, *H. pylori* have evolved multiple mechanisms to adapt in humans. In children, the infection is largely asymptomatic, rarely creates complications, and elicits reduced inflammation and an immune response that is tolerogenic to allow for bacterial persistence over many years. The persistent infection favors the potential for spread to others who more often share key risk factors, such as low socioeconomic status, having a mother who is infected, having multiple siblings, living in crowded conditions, and drinking untreated water.

*H. pylori* has mechanisms that allow survival in one of the harshest environments in the body, such as the highly acidic conditions in the stomach. The expression of urease allows the bacteria to raise the local pH, and this, in turn, represents an obvious advantage for the long-term colonization of *H. pylori* in the stomach. Although the infection leads to a marked inflammatory response with the infiltration of T lymphocytes in the gastric mucosa, the immune response is misguided and is ineffective in clearing the infection. Multiple virulence factors expressed by the bacteria contribute to immune evasion either by inhibiting antigen processing, T cell activation, and proliferation or by affecting the expression of an important family of receptors that determine T cell activation and influence T cell phenotype.

In contrast to childhood infections, the infection in adults may lead to clinically significant outcomes that include peptic ulcer disease, MALT, or gastric cancer. Most gastric cancer patients are > 50 years of age. However, a recent study found that in the United States there is an increasing incidence of cases of non-cardia gastric cancer in people < 50 years of age^151^. It is important to note that non-cardia gastric cancer is often diagnosed at a metastatic stage and is difficult to treat. As a pathogen-associated form of cancer, gastric cancer is potentially preventable with a vaccine. For the development of an effective vaccine, it is necessary to understand the spectrum of *H. pylori*-associated disease in children, why their gastric immune response to *H. pylori* differs from that of adults, improve our understanding of the immunopathogenesis of gastric disease outcomes linked to *H pylori*, and identify candidate targets that will elicit an immune response that is not affected by any of the known *H. pylori* immune evasion mechanisms.
